# Supplementary figures and images for: Carvedilol improves glucose tolerance and insulin sensitivity in treatment of adrenergic overdrive in high fat diet-induced obesity in mice
Source: PLoS One. 2019 Nov 4;14(11):e0224674. doi: 10.1371/journal.pone.0224674 (PMC6827914; doi:10.1371/journal.pone.0224674)

**Figure S1**

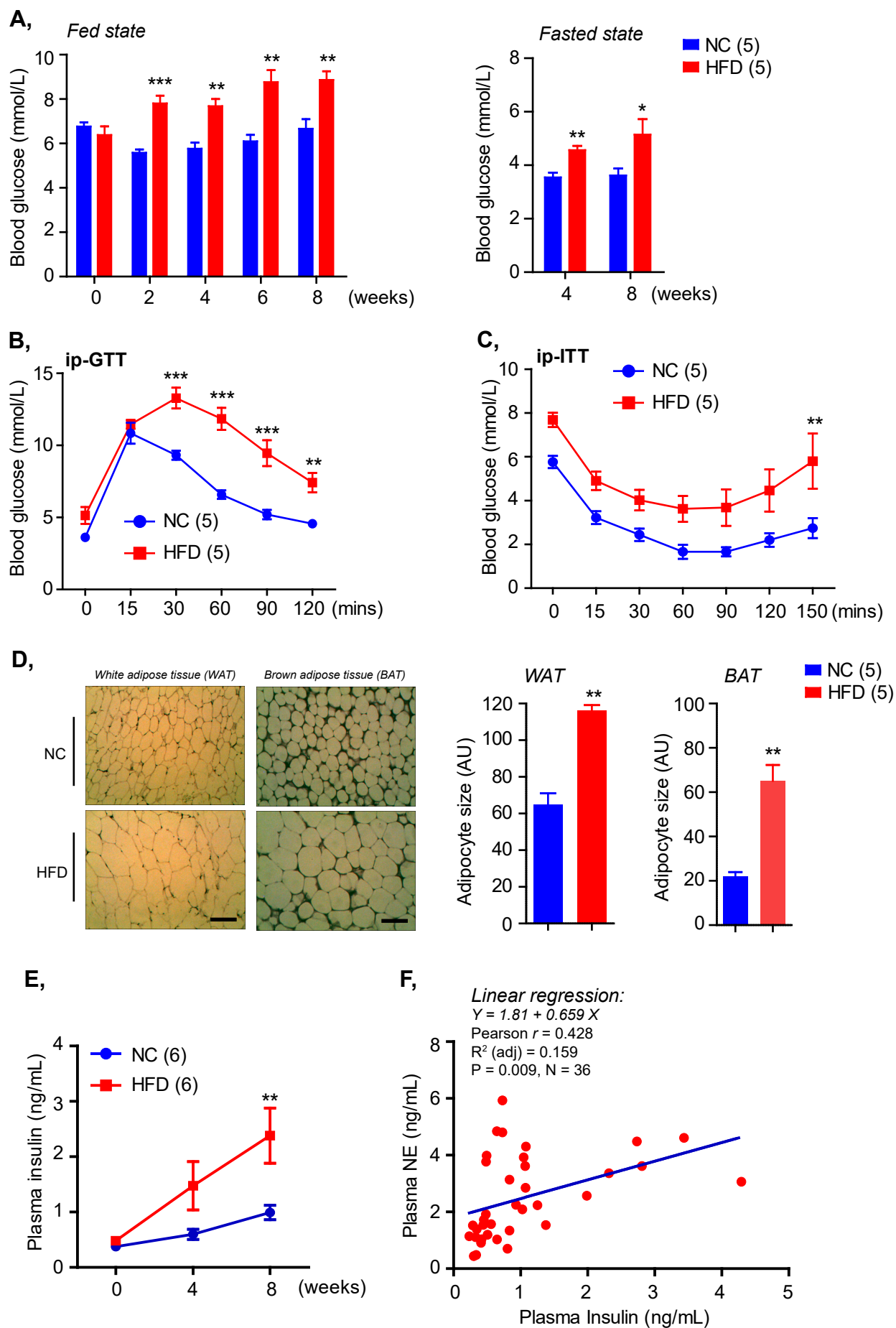

Supplement: S1 Fig — A, Blood glucose levels at fed (left) and fasted state (right) of mice fed NC or HFD. B, C, GTT (B) and ITT (C) of mice performed after 4 weeks feeding NC or HFD. D, Representative figures of H&E staining (left) and quantification of adipocyte size (right) of white and brown adipose tissues of mice fed NC or HFD. Scale bar, 100 μm in WAT and 50 μm in BAT. E, Plasma insulin levels of mice during 8-week period feeding NC or HFD. F, Linear correlation analysis of basal plasma norepinephrine and insulin concentrations during HFD-induced obesity. Data are presented as mean ± S.E.M. Student’s t-tests in bar graphs and two-way ANOVA with Bonferroni’s post-tests in line graphs. *P< 0.05, **P<0.01, ***P<0.001. Hormone measurement and histological analysis were performed in triplicate. (PDF) [file pone.0224674.s001.pdf]

**A,**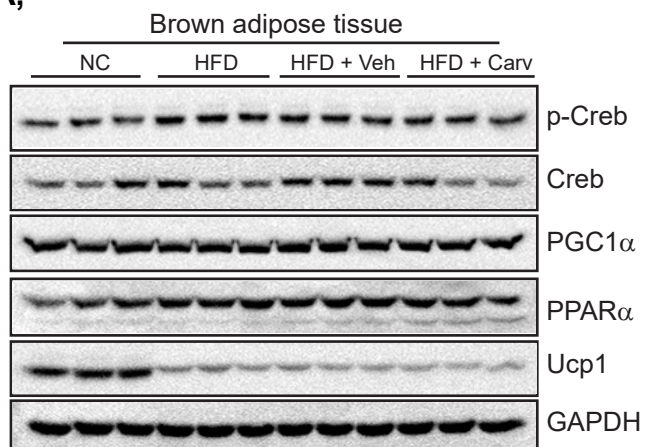**B,**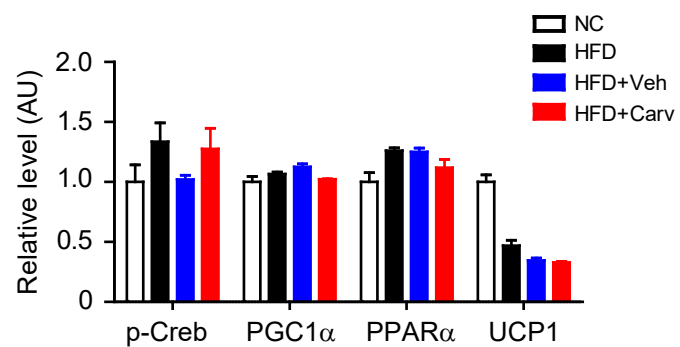**C,**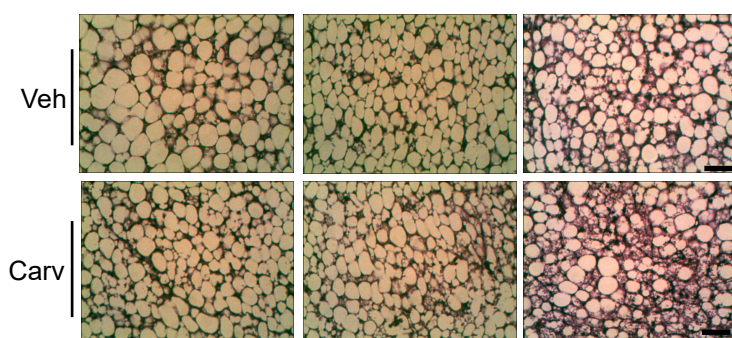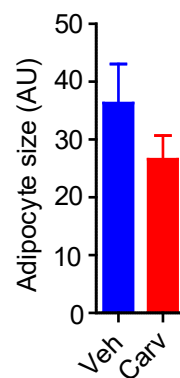

Supplement: S2 Fig — A, B, Immunoblots and quantitative densitometry (B) showing the levels of p-Creb, PGC1α, PPARα and UCP1 in BAT samples of NC-fed mice and HFD-fed mice treated with vehicle (Veh) or carvedilol (Carv). Normalized to total Creb or GAPDH. C, Representative figures of H&E staining (left) and quantification of brown adipocyte size (right) of HFD-fed mice treated with vehicle or carvedilol. Scale bar, 50 μm. Data are presented as mean ± S.E.M. Student’s t-tests or one-way ANOVA with Turkey’s post-tests in bar graphs. *P<0.05. Western blot and histological analyses were performed in triplicate. (PDF) [file pone.0224674.s002.pdf]

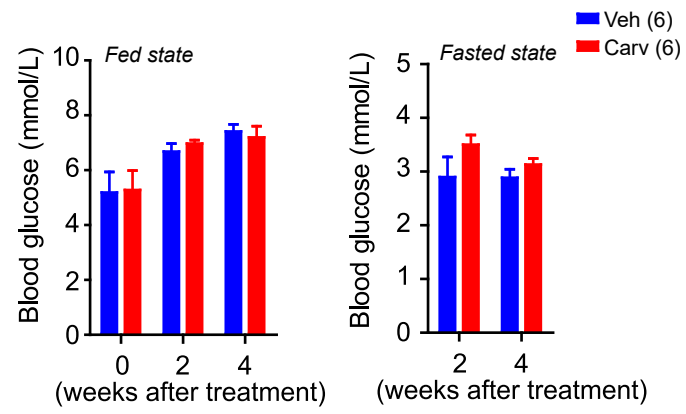

Supplement: S3 Fig — Blood glucose levels at fed state (left) and fasted state (right) of HFD-fed mice treated with vehicle or carvedilol. Data are presented as mean ± S.E.M. Student’s t-tests. (PDF) [file pone.0224674.s003.pdf]

## Liver samples

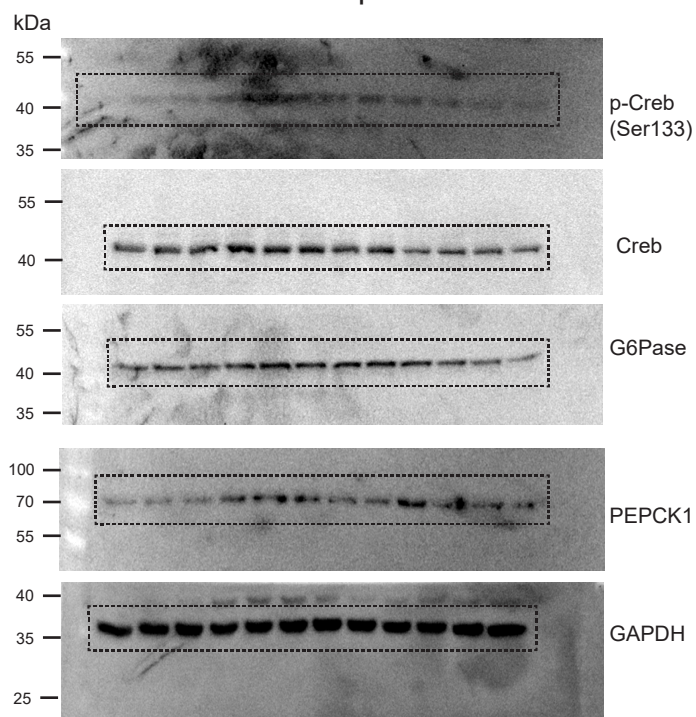

## Muscle samples

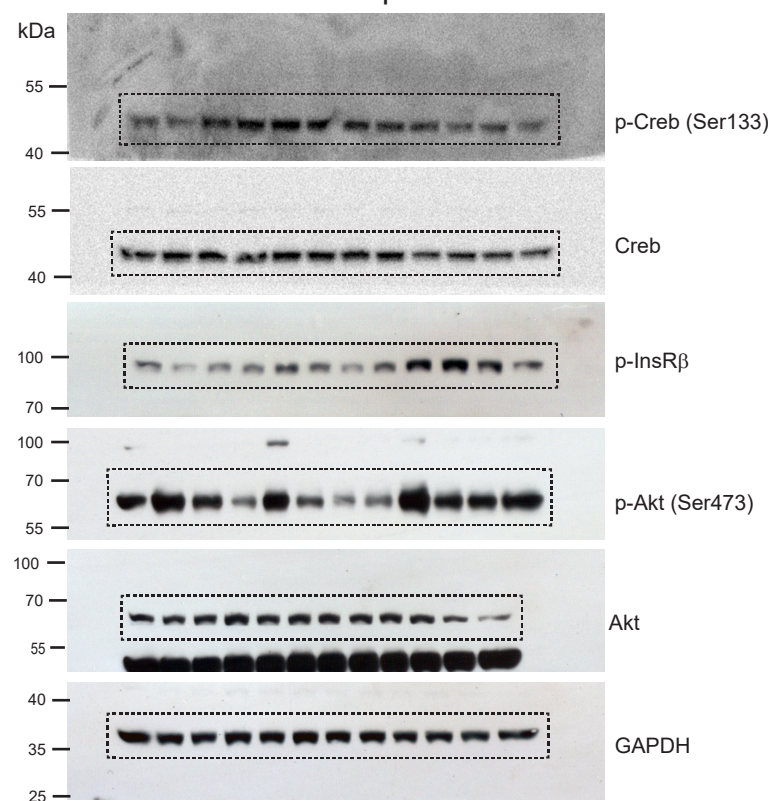

## BAT samples

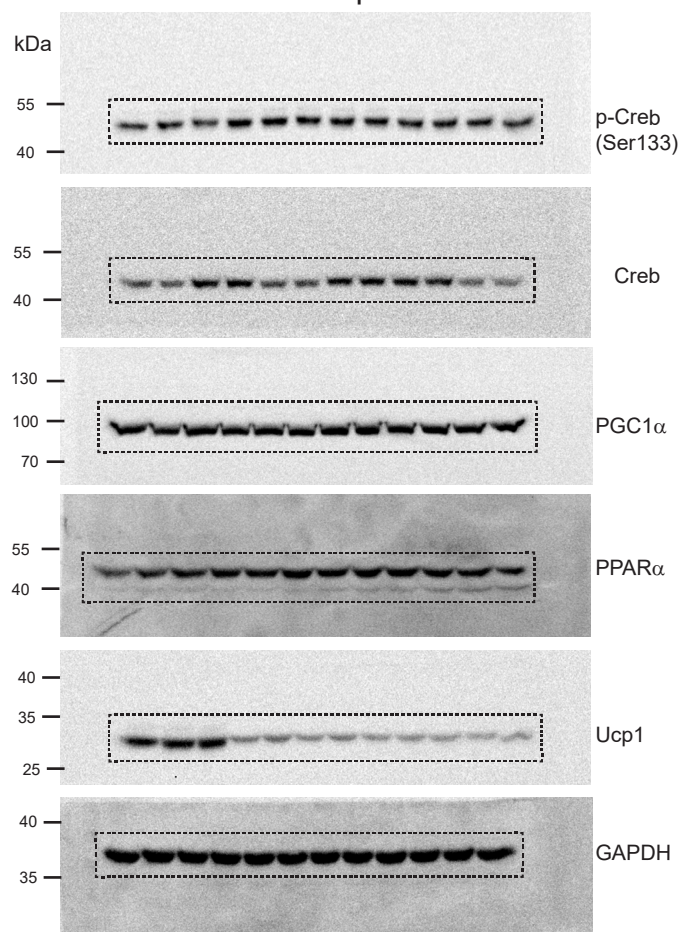

Supplement: S4 Fig — (PDF) [file pone.0224674.s004.pdf]
