# Supplementary material for: Carvedilol improves glucose tolerance and insulin sensitivity in treatment of adrenergic overdrive in high fat diet-induced obesity in mice
Source: PLoS One. 2019 Nov 4;14(11):e0224674. doi: 10.1371/journal.pone.0224674 (PMC6827914; doi:10.1371/journal.pone.0224674)
Supplement: Attachment — Submitted filename: Response to Reviewers.doc [file pone.0224674.s005.doc]

September 27, 2019

**Rebuttal Letter** (PONE-D-19-21383)

We sincerely appreciate the Academic Editor and Reviewers for taking your valuable time to consider our work. Here, we would like to address the journal requirements from the academic editor and the reviewer’s comments as follows:

**Journal Requirements:**

1. Please ensure that your manuscript meets PLOS ONE's style requirements, including those for file naming. The PLOS ONE style templates can be found at http://www.journals.plos.org/plosone/s/file?id=wjVg/PLOSOne_formatting_sample_main_body.pdf and http://www.journals.plos.org/plosone/s/file?id=ba62/PLOSOne_formatting_sample_title_authors_affiliations.pdf

**Response:**

We have formatted the manuscript following PLOS ONE's style requirements. Additionally, for this reason, we have rearranged the Figure 3 and Supplemental Figure 3 to make 04 main figures in the revised manuscript. We have therefore updated the Figs 3 and 4 and S3 Fig and the text in the “Results” and “Figure legends” sections in the revised manuscript correspondingly (lines 273-291, 537-553 and 584-587).

2. During your revisions, please note that a simple title correction is required: "Carvedilol Improves Glucose Tolerance and Insulin Sensitivity in Treatment of Adrenergic Overdrive in High Fat Diet-induced Obesity *in mice*". Please ensure this is updated both in the manuscript file and the online submission information.

**Response:**

We have corrected and updated the title “Carvedilol improves glucose tolerance and insulin sensitivity in treatment of adrenergic overdrive in high fat diet-induced obesity in mice” both in the revised manuscript (line 2) and the online submission information as the Academic Editor suggested.

We sincerely appreciate the Academic Editor for this insightful correction to better clarify our findings.

3. To comply with PLOS ONE submissions requirements, in your Methods section, please provide additional information on the animal research and ensure you have included details on (i) methods of sacrifice, (ii) methods of anesthesia and/or analgesia, and (iii) efforts to alleviate suffering.

**Response:**

We apologize the Academic Editor for the missing information of animal research on (i) methods of sacrifice, (ii) methods of anesthesia and/or analgesia, and (iii) efforts to alleviate suffering in the “Methods” section. We sacrificed mice by decapitation after anesthetizing with ketamine. We have provided this detail information in the “Methods” section in the revised manuscript (lines 131-132).

4. PLOS ONE now requires that authors provide the original uncropped and unadjusted images underlying all blot or gel results reported in a submission’s figures or Supporting Information files. This policy and the journal’s other requirements for blot/gel reporting and figure preparation are described in detail at https://journals.plos.org/plosone/s/figures#loc-blot-and-gel-reporting-requirements and https://journals.plos.org/plosone/s/figures#loc-preparing-figures-from-image-files. When you submit your revised manuscript, please ensure that your figures adhere fully to these guidelines and provide the original underlying images for all blot or gel data reported in your submission. See the following link for instructions on providing the original image data: https://journals.plos.org/plosone/s/figures#loc-original-images-for-blots-and-gels.

In your cover letter, please note whether your blot/gel image data are in Supporting Information or posted at a public data repository, provide the repository URL if relevant, and provide specific details as to which raw blot/gel images, if any, are not available. Email us at plosone@plos.org if you have any questions.

**Response:**

We have provided the original uncropped and unadjusted images of all blots/gels in the S4 Fig of the Supporting Information file.

**Reviewers' comments:**

Reviewer's Responses to Questions

**Comments to the Author**

1. Is the manuscript technically sound, and do the data support the conclusions?

The manuscript must describe a technically sound piece of scientific research with data that supports the conclusions. Experiments must have been conducted rigorously, with appropriate controls, replication, and sample sizes. The conclusions must be drawn appropriately based on the data presented.

Reviewer #1: Yes

Reviewer #2: Yes

**Response:**

We sincerely appreciate the reviewer’s comment and suggestion on our manuscript.

2. Has the statistical analysis been performed appropriately and rigorously?

Reviewer #1: Yes

Reviewer #2: Yes

**Response:**

We sincerely appreciate the reviewer’s comment and suggestion on our manuscript.

3. Have the authors made all data underlying the findings in their manuscript fully available?

The PLOS Data policy requires authors to make all data underlying the findings described in their manuscript fully available without restriction, with rare exception (please refer to the Data Availability Statement in the manuscript PDF file). The data should be provided as part of the manuscript or its supporting information, or deposited to a public repository. For example, in addition to summary statistics, the data points behind means, medians and variance measures should be available. If there are restrictions on publicly sharing data—e.g. participant privacy or use of data from a third party—those must be specified.

Reviewer #1: Yes

Reviewer #2: Yes

**Response:**

We sincerely appreciate the reviewer’s comment and suggestion on our manuscript.

4. Is the manuscript presented in an intelligible fashion and written in standard English?

PLOS ONE does not copyedit accepted manuscripts, so the language in submitted articles must be clear, correct, and unambiguous. Any typographical or grammatical errors should be corrected at revision, so please note any specific errors here.

Reviewer #1: Yes

Reviewer #2: Yes

**Response:**

We sincerely appreciate the reviewer’s comment and suggestion on our manuscript.

**5. Review Comments to the Author**

**Reviewer #1:**

In the manuscript entitled “Carvedilol Improves Glucose Tolerance and Insulin Sensitivity in Treatment of Adrenergic Overdrive in High Fat Diet-induced Obesity”, Nguyen et al. have investigated the metabolic effects of carvedilol, a third-generation beta-blocker agent, in the HFD-induced obese mice in which they found that the basal plasma norepinephrine of those mice is chronically elevated and correlated to the plasma leptin concentrations. They showed that treatment of adrenergic overactivation from the excessive norepinephrine in HFD-fed mice with carvedilol enhances glucose tolerance and insulin sensitivity by blocking the glucose overproduction in the liver and increasing muscular insulin sensitivity. Overall, the manuscript appears very solid and well done. The manuscript would benefit from addressing following points:

(1) The p-Creb level should be assessed together with total Creb level in addition to the loading protein in the samples.

**Response:**

We agree with the reviewer that the p-Creb level should be measured together with total Creb. We have therefore run the samples to check total Creb levels and provided the results in the Figs 2A, F and S2A Fig in the revised manuscript. Similar to the loading protein GAPDH in the samples, the levels of total Creb were comparable between control and carvedilol treatment groups. Further, we have updated the Figs 2A-B, F-G and S2A and B Fig using the ratios of p-Creb/total Creb correspondingly. We thank the reviewer for this insightful suggestion.

(2) In Figure 2B, G and Figure S2B, the unit of Y-axis should be provided.

**Response:**

We apologize the reviewer for the missing information on the unit of Y-axis in the Figure 2B, G and Figure S2B. We have updated the Fig 2B, G and S2B Fig providing the unit of Y-axis in the revised manuscript. We sincerely thank the reviewer for this comment.

(3) In Figure S1D, the scale bar does not correspond to the absolute readings in the bar graphs. The authors should revise it.

**Response:**

We apologize the reviewer for the confusing correspondence between scale bar and absolute readings in the bar graphs in Figure S1D. We have revised the quantification of histological images and updated the results in the S1D Fig in the revised manuscript correspondingly. We sincerely appreciate the reviewer for this insightful comment to improve our manuscript.

**Reviewer #2:**

This study submitted by Nguyen et al. claims that adrenergic overdrive such as high level of catecholamine link to plasma leptin level, consequently it contributes to the genesis of metabolic disorder. Nguyen et al. found that basal norepinephrine level is increased with a strong correlation of plasma leptin concentration in high-fat diet (HFD)-induced obese mouse model. By treating carvedilol, a third-generation beta type blocker, in HFD-induced obese mouse, the glucose tolerance and insulin sensitivity were improved.

This study is well designed and formed. Particularly Nguyen et al. carefully propose important point regarding potential causation of metabolic disorder which is the correlation between adrenergic overdrive and plasma leptin level, and they verified the effect of carvedilol against adrenergic overdrive.

I have an only minor comment regarding this study.

Figure legend should describe how many (N=??) repeat each experiment regardless of its type including western blot and its analysis. Although figure itself has displayed a number which I assume is an experimental number.

**Response:**

We apologize the reviewer for missing information on the experimental replication in the figure legends. We performed the experiments of hormone measurement, histological and western blot analyses in triplicate. We have updated the figure legends to describe information on the replication of each experiment in the revised manuscript correspondingly (lines 518, 534-535, 546, 569-570 and 581-582). We appreciate the reviewer for this insightful and constructive suggestion.

6. PLOS authors have the option to publish the peer review history of their article (what does this mean?). If published, this will include your full peer review and any attached files.

If you choose “no”, your identity will remain anonymous but your review may still be made public.

Do you want your identity to be public for this peer review? For information about this choice, including consent withdrawal, please see our Privacy Policy.

Reviewer #1: No

Reviewer #2: No

**Response:**

We sincerely appreciate the reviewer’s comment and suggestion on our manuscript.

We sincerely thank the reviewers for the careful reading and insightful comments on our manuscript.

Sincerely,

Khanh V. Doan, Ph.D.,

Department of Pharmacology,

School of Medicine, Tan Tao University.

Address: Tan Tao University Ave., Tan Duc E.City, Duc Hoa, Long An 850000, Viet Nam.

Tel: (+84-272)-376-9216

Fax: (+84-272)-376-9208

Cell: (+84-91)-185-1177

Email: doankhanh.pharm@gmail.com
